# Zinc‐finger E‐box‐binding homeobox 1 (ZEB1) plays a crucial role in the maintenance of lung cancer stem cells resistant to gefitinib

**DOI:** 10.1111/1759-7714.13937

**Published:** 2021-03-25

**Authors:** Fariz Nurwidya, Fumiyuki Takahashi, Wira Winardi, Ken Tajima, Yoichiro Mitsuishi, Akiko Murakami, Isao Kobayashi, Takeshi Nara, Muneaki Hashimoto, Motoyasu Kato, Moulid Hidayat, Kentaro Suina, Daisuke Hayakawa, Tetsuhiko Asao, Ryo Ko, Takehito Shukuya, Toshifumi Yae, Naoko Shimada, Yasuko Yoshioka, Shinichi Sasaki, Kazuhisa Takahashi

**Affiliations:** ^1^ Department of Respiratory Medicine Juntendo University, Graduate School of Medicine Tokyo Japan; ^2^ Research Institute for Diseases of Old Ages, Juntendo University Graduate School of Medicine Tokyo Japan; ^3^ Department of Molecular and Cellular Parasitology Juntendo University, Graduate School of Medicine Tokyo Japan; ^4^ Faculty of Pharmacy Iryo Sosei University Fukushima Japan; ^5^ Health and Medical Research Institute, National Institute of Advanced Industrial Science and Technology (AIST) Kagawa Japan; ^6^ Division of Gene Regulation Institute for Advanced Medical Research, Keio University School of Medicine Tokyo Japan; ^7^ Leading Center for the Development and Research of Cancer Medicine, Juntendo University Graduate School of Medicine Tokyo Japan

**Keywords:** cancer stem cells, epithelial‐mesenchymal transition, gefitinib resistance, lung cancer, ZEB1

## Abstract

**Background:**

Zinc‐finger E‐box‐binding homeobox 1 (ZEB1) is an important regulator of epithelial‐mesenchymal transition (EMT) and is involved in the maintenance of cancer stem cells (CSCs) via miR‐200c and BMI1 pathway. Recent studies revealed that ZEB1 contributes to the EMT‐mediated acquired resistance to gefitinib in *EGFR*‐mutant non‐small cell lung cancer (NSCLC). However, the precise role of ZEB1 in the maintenance of lung CSCs that lead to acquired resistance to gefitinib remains unclear.

**Methods:**

PC9 and HCC827 NSCLC cell lines were treated with high concentrations of gefitinib, and surviving cells were referred to as “gefitinib‐resistant persisters” (GRPs). ZEB1 knockdown or overexpression was performed to determine the biological significance of ZEB1 in the CSC features of GRPs, and animal models were studied for in vivo validation. Expression of ZEB1, BMI1, and ALDH1A1 was analyzed by immunohistochemistry in tumor specimens from NSCLC patients with acquired resistance to gefitinib.

**Results:**

GRPs had characteristic features of mesenchymal and CSC phenotypes with high expression of ZEB1 and BMI1, and decreased miR‐200c, in vitro and in vivo. ZEB1 silencing attenuated the suppression of miR‐200c, resulting in the reduction in BMI1 and reversed the mesenchymal and CSC features of GRPs. Furthermore, ZEB1 overexpression induced EMT and increased the levels of CD133‐ and BMI1‐positive GRPs in vitro and gefitinib resistance in vivo. Finally, ZEB1, BMI1, and ALDH1A1 were highly expressed in tumor specimens from *EGFR*‐mutant NSCLC patients with gefitinib resistance.

**Conclusions:**

ZEB1 plays an important role in gefitinib‐resistant lung CSCs with EMT features via regulation of miR‐200c and BMI1.

## Graphical abstract

Gefitinib‐resistant persisters (GRPs) of *EGFR*‐mutant NSCLC cells have characteristic features of mesenchymal and CSC phenotypes. ZEB1 is highly expressed in GRPs and involved in the maintenance of lung CSCs resistant to gefitinib via miR‐200c‐BMI1 pathway.

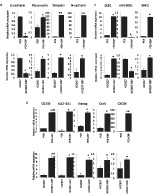

## INTRODUCTION

Progression‐free survival (PFS) in patients with epidermal growth factor receptor (*EGFR*)‐mutant non‐small cell lung cancer (NSCLC) is limited due to acquired resistance to EGFR tyrosine kinase inhibitors (TKIs) such as gefitinib,^1^ and investigations of the molecular mechanisms underlying resistance to EGFR‐TKI are performed globally. The most well‐known mechanisms of acquired resistance are *EGFR* T790M secondary mutation, *MET* amplification, overexpression of HGF, mutation or amplification of *HER2*, transformation to small cell lung cancer, and *PIK3CA* mutation.^2^, ^3^, ^4^, ^5^, ^6^, ^7^, ^8^ However, 30% of the mechanisms of acquired resistance to EGFR‐TKIs are not completely understood.^7^

Cancer stem cells (CSCs), also known as tumor‐initiating cells and stem‐like cancer cells, express stem cell markers such as CD133,^9^ BMI1,^10^ ALDH1A1,^11^ Oct4,^12^ and CXCR4^13^ in NSCLC. Several recent reports have suggested the involvement of lung CSCs in resistance to EGFR‐TKI in NSCLC.^13^, ^14^ Accumulating evidence also suggests that the generation of CSCs is associated with epithelial‐mesenchymal transition (EMT),^15^ which promotes malignant tumor progression. Moreover, EMT is also involved in resistance to EGFR‐TKI.^16^, ^17^, ^18^, ^19^

Zinc‐finger E‐box‐binding homeobox 1 (ZEB1) is a crucial EMT inducer in various human cancers.^20^ Moreover, ZEB1 is also associated with stemness maintenance by repressing stemness‐inhibiting microRNAs, including miR‐200c.^21^ A recent study reported that ZEB1 is involved in EMT‐mediated acquired resistance to EGFR‐TKIs.^22^, ^23^ However, the role of ZEB1 in the maintenance of lung CSCs that lead to acquired resistance to gefitinib is unclear.

In this study, using a short duration of treatment with high concentration of gefitinib, we established a unique model to obtain gefitinib‐resistant persisters (GRPs) of *EGFR*‐mutant NSCLC with stem cell features, in vitro as well as in vivo. We found that ZEB1 was highly expressed in the GRPs model and played important roles in the induction of EMT and maintenance of CSCs that eventually led to acquired resistance to gefitinib. We also integrated these in vitro and in vivo findings with clinical data from *EGFR*‐mutant NSCLC patients who experienced relapse after initial response to gefitinib.

## METHODS

### Cells, reagents, and establishment of ZEB1‐silenced and ZEB1‐overexpressed lung cancer cell lines

PC9 cells were kindly provided by Dr Kazuto Nishio (Department of Genome Biology, School of Medicine, Kindai University, Osaka). HCC827 cells were purchased from the American Type Culture Collection (Manassas). Cell lines were verified to be mycoplasma‐free. Gefitinib was purchased from JS Research Chemicals Trading.

For a stable knockdown of ZEB1 (shZEB1), following sequences were annealed and cloned into the pLKO1‐puro vector: shZEB1 forward, 5′‐ CCGGGCAACAATACAAGAGGTTAAACTCGAGTTTAACCTCTTGTATTGTTGCTTTTTG‐3′; shZEB1 reverse 5′‐AATTCAAAAAGCAACAATACAAGAGGTTAAACTCGAGTTTAACCTCTTGTATTGTTGC‐3′. The control plasmid was MISSION nontarget shRNA control vector (Cat. Number SHC002, Sigma‐Aldrich). Constructs were transfected into the lentivirus‐producing 293FT cells and lentivirus‐containing supernatant was transduced into the indicated cell lines followed by selection using puromycin (Invitrogen). To avoid off‐target effects, transient knockdown of ZEB1 was also performed in PC9 cells (see Figure S2).

Lentiviral vector expressing ZEB1 was kindly provided by Dr Shyamala Maheswaran (Center for Cancer Research, Massachusetts General Hospital and Harvard Medical School, Boston, USA). A cDNA encoding human ZEB1, conjugated with flag protein sequences, was cloned into the pLenti4 vector. Lentivirus packaging 293FT cells were transfected with the pLenti4‐flag‐ZEB1 plasmid or pLenti4‐V5DEST using the ViraPower Lentiviral Expression System (Invitrogen); and, the lentiviruses released into the culture supernatant were transduced into PC9 and HCC827 cells, followed by selection with zeocin (Invitrogen) to obtain stable PC9‐ZEB1 and HCC827‐ZEB1 cells. The ZEB1‐silencing and ZEB1‐overexpression plasmid transfections were performed using Lipofectamine LTX and PLUS reagents (Invitrogen).

Generation of GRPs of PC9 and HCC827, quantitative PCR, immunofluorescence, and sphere formation assays were performed as previously described.^24^ Light microscopy, western blotting, list of employed antibodies, small interfering RNA transfection, and list of qPCR primers, are described in Supplementary Materials and Methods S1.

### Xenograft studies in NOG mice

NOD/Shi‐scid/IL‐2Rcnull (NOG) mice (7‐week‐old, female) were purchased from the Central Institute for Experimental Animals (Kanagawa, Japan). All mice were shipped to Juntendo University and handled under pathogen‐free conditions. The mice were housed in a room under controlled temperature (25°C), humidity, and lighting (12 h light/dark cycle). To determine the in vivo tumor growth of GRPs, 1 × 10^2^ PC9 and PC9‐GRPs cells were mixed with Matrigel (BD Biosciences) and injected into both the flanks of NOG mice. Tumor formation was evaluated, and tumor size was measured until 44 days after injection. Tumor volume was calculated using the following formula: volume (mm^3^) = 0.5 × length × width^2^. The same protocol was employed for the GRPs of PC9‐shZEB1. The tumor samples were divided for mRNA analysis and paraffin‐fixed for fluorescence immunohistochemistry (F‐IHC). Further, for the in vivo ZEB1‐induced resistance study, 1 × 10^5^ PC9‐mock and PC9‐ZEB1 cells were transplanted subcutaneously in the mice. Tumor growth was monitored daily, and when the solid tumors reached a volume of 75 mm^3^, the animals were assigned to receive vehicle, 10 mg/ml of gefitinib, or 20 mg/ml of gefitinib for 14 days.

### Ethics

All animal experiments were performed in accordance with the Fundamental Guidelines for Proper Conduct of Animal Experiment and Related Activities in Academic Research Institutions under the jurisdiction of the Ministry of Education, Culture, Sports, Science and Technology of Japan (Notice No. 71, 2006), and were approved by the Committee for Animal Experimentation of Juntendo University (Approval No. 240182).

### Patient sample collection

Tumor samples were obtained from patients with lung cancer who visited the Juntendo University Hospital. Patients gave their consent prior to enrollment in the study under Juntendo University Institutional Review Board (IRB)‐approved protocols. All experiments conformed to the principles outlined in the WMA Declaration of Helsinki.

### Fluorescence immunohistochemistry (F‐IHC)

Immunohistochemistry for ZEB1, BMI1, and ALDH1A1 was performed as described in Supporting methods. Double‐staining was performed using an antibody specific either to *EGFR*‐19del or *EGFR*‐L858R depending on the *EGFR* mutational status of the patient. Percentages of ZEB1‐, BMI1‐, or ALDH1A1‐positive cells in DAPI staining were graded as follows: −/negative expression (0%–5%); +/weak expression (5.1%–33%); ++/moderate expression (33.1%–66%); +++/strong expression (66.1%–100%).

### Statistical analysis

Statistical analyses were performed using GraphPad Prism 6.0 (GraphPad Software). Values were compared using a two‐tailed Student's *t*‐test. Differences between the means were considered statistically significant at *p* < 0.05.

## RESULTS

### GRPs have characteristic gene expression profiles of mesenchymal and CSCs phenotype and high ZEB1 expression in NSCLC cell lines

The NSCLC cell lines PC9 and HCC827, which harbor an activating *EGFR* mutation, were exposed to high concentrations of gefitinib (1 μM for PC9 and 2 μM for HCC827). Nine days after exposure to gefitinib, a small fraction of viable cells survived and remained, whereas most of the cells died within a few days. These surviving PC9 and HCC827 cells were termed GRPs (PC9‐GRPs and HCC827‐GRPs, respectively). GRPs were extremely quiescent, and further isolated for gene expression analysis. We have previously demonstrated that short tandem repeat (STR) profiles of the parental cells and GRPs were identical by analysis of genomic DNA, indicating that GRPs did not arise from contaminating cells.^24^ Furthermore, neither the *EGFR* T790M mutation nor *MET* gene amplification was observed in PC9‐ or HCC827‐GRPs.^24^

**FIGURE 1:**
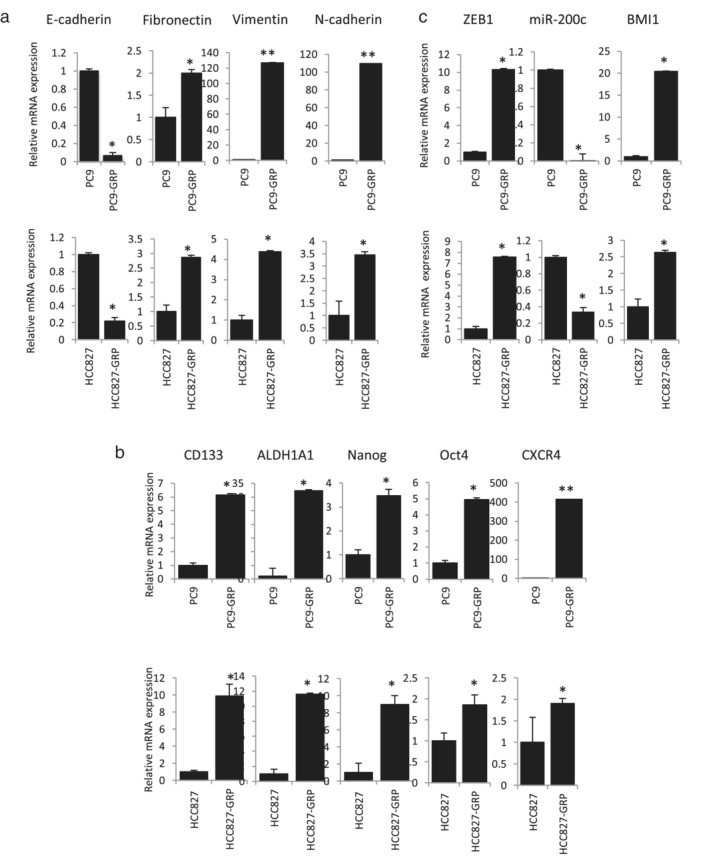
Gefitinib‐resistant persisters (GRPs) of PC9 and HCC827 show gene signatures reminiscent of epithelial‐mesenchymal transition (EMT) and cancer stem cells (CSCs). (a) The mRNA expression of E‐cadherin, fibronectin, vimentin, and N‐cadherin is evaluated by quantitative real‐time PCR (qPCR). (b) The mRNA expression of stem cell‐related factors is evaluated by qPCR. (c) The mRNA expression of zinc‐finger E‐box‐binding homeobox 1 (ZEB1) and BMI1, and miRNA expression of miR‐200c is evaluated by qPCR. Data are normalized to beta actin (ACTB) expression. All values are average of triplicate experiments, with error bars indicating SEM (*p < 0.05; **p < 0.01)

To identify the mechanism underlying gefitinib resistance in the cell line model, we first investigated the expression of EMT‐ and CSC‐related genes in parental cells and GRPs. The qPCR analysis indicated loss of epithelial marker E‐cadherin, and increased expression of mesenchymal markers including fibronectin, vimentin, and N‐cadherin in PC9‐ and HCC827‐GRPs than in parental cells (Figure 1(a)). Furthermore, CSC genes such as CD133, ALDH1A1, Nanog, Oct4, and CXCR4 were highly expressed in PC9‐GRPs and HCC827‐GRPs than in parental cells (Figure 1(b)). These findings suggest that the small population of NSCLC cells that were highly enriched for EMT and CSC‐related gene expression could survive and remain viable upon treatment with gefitinib.

ZEB1 is associated with EMT induction and maintenance of CSCs by repressing miR‐200c, which can target BMI1, a known regulator of stemness.^10^, ^21^, ^25^ As shown in Figure 1(c), ZEB1 was highly expressed in both PC9‐ and HCC827‐GRPs than in parental cells. Interestingly, expression of miR‐200c was downregulated and BMI1 was upregulated in both GRPs than in parental cells (Figure 1(c)). These findings suggest a role for ZEB1 in the persistence of the mesenchymal and CSC genotypes that are resistant to gefitinib in *EGFR* mutation‐positive NSCLC via the ZEB1‐miR200c‐BMI1 axis.

### GRPs maintain mesenchymal and CSC genotypes and high ZEB1 expression in vivo

**FIGURE 2:**
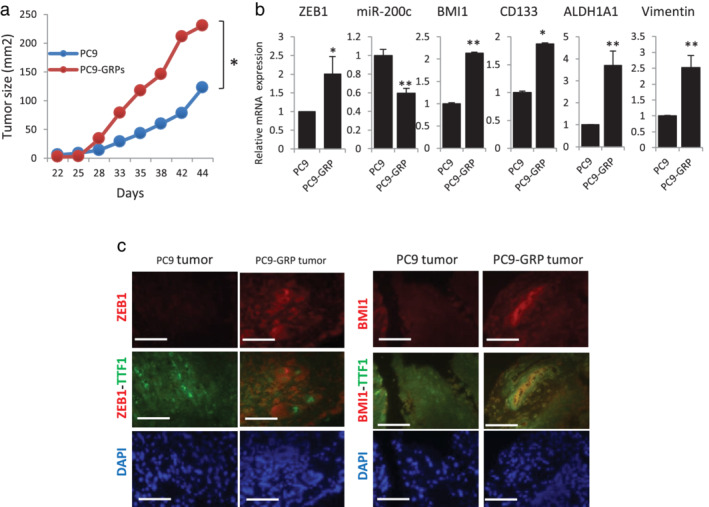
PC9‐GRP tumors show increased tumor growth, and high gene expression of zinc‐finger E‐box‐binding homeobox 1 (ZEB1), low miR‐200c, high BMI1, and gene expression profiles reminiscent of epithelial‐mesenchymal transition (EMT) and cancer stem cells (CSCs). (a) Comparison of tumor size (volume) between PC9 and PC9‐GRPs in vivo. (b) Quantitative real‐time PCR (qPCR) analysis of expression of ZEB1, miR‐200c, BMI1, CD133, ALDH1A1, and vimentin. Data are normalized to expression of beta actin (ACTB) and represent mean ± SEM for at least three tumors (*p < 0.05; **p < 0.01). (c) Double‐staining fluorescence immunohistochemistry for thyroid transcription factor 1 (TTF1) to distinguish cancer tissue from noncancer tissue and evaluate the expression of ZEB1 and BMI1 in PC9‐GRPs and PCR9‐parent tumors. Scale bars indicate 200 μm. GRP, gefitinib‐resistant persisters

To investigate whether PC9‐GRPs maintain the gene expression profile of ZEB1‐miR200c‐BMI1 axis as well as mesenchymal and CSC genotypes in vivo, we injected 100 cells of PC9‐parent and PC9‐GRPs into both the flanks of NOG mice. Tumor growth of PC9‐GRPs was significantly faster than that of PC9‐parent cells (Figure 2(a)). PC9‐GRPs tumors showed lower expression of miR‐200c, and higher expression of ZEB1, BMI1, and other CSC‐related genes such as CD133, ALDH1A1 and mesenchymal marker vimentin than PC9‐parent tumors (Figure 2(b)). We also examined protein expression of ZEB1 and BMI1 by IHC. Thyroid transcription factor 1 (TTF1) staining was also used to confirm presence of cancer cells in the tumor specimens. As shown in Figure 2(c), both ZEB1 and BMI1 were highly expressed in cancer cells with TTF1 staining in PC9‐GRPs tumors than that in PC9‐parent tumors. These findings indicated that PC9‐GRPs maintained the gene expression profile of ZEB1‐miR200c‐BMI1 axis as well as mesenchymal and CSC genotypes in vivo.

Further, to confirm this expression profile in gefitinib‐resistant tumors in vivo, we transplanted PC9 cells into NOG mice (Figure S1). When the tumor volume reached approximately 75 mm^3^, as measured with digital calipers, tumor‐bearing mice were treated with gefitinib (20 mg/kg) via intraperitoneal injection (five times/week). After 14 days of gefitinib treatment, the tumors remained, and these tumors were referred to as gefitinib‐resistant tumors (GRTs). PC9‐GRTs retained lower expression of miR‐200c, and higher expression of ZEB1, BMI1, and CD133, ALDH1A1, Nanog, CXCR4 and vimentin than PC‐9 parent tumors (Figure S1). These in vivo findings of PC9‐GRTs were consistent with those of PC9‐GRPs transplanted in NOG mice.

### Silencing ZEB1 reversed mesenchymal and CSC features in GRPs

**FIGURE 3:**
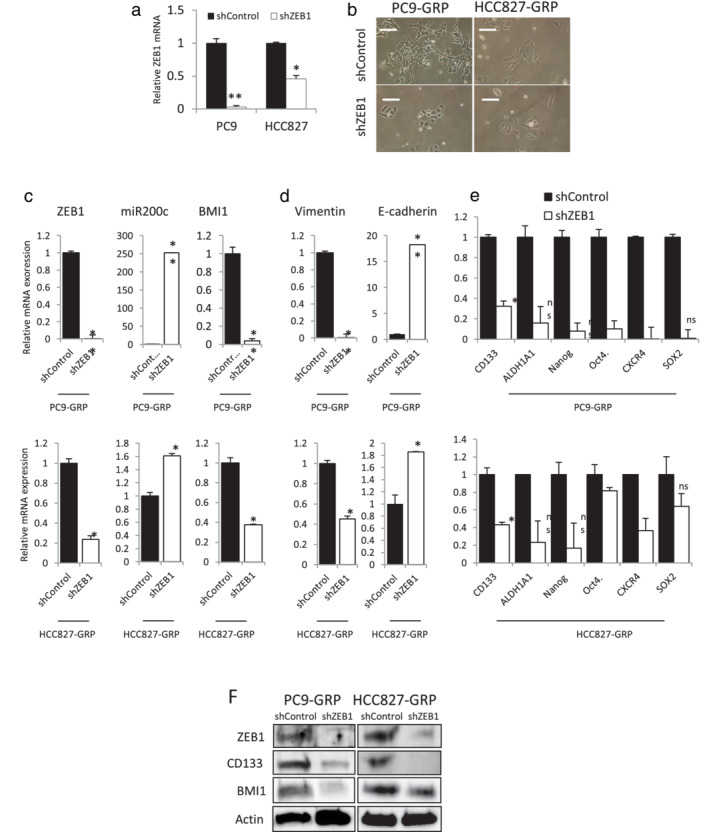
Stable knockdown of zinc‐finger E‐box‐binding homeobox 1 (ZEB1) reverses epithelial‐mesenchymal transition (EMT) and cancer stem cells (CSC) features in PC9‐ and HCC827‐GRPs. (a) Quantitative real‐time PCR (qPCR) analysis for confirming knockdown efficiency of ZEB1. (b) Microscopic images for ZEB1‐silenced PC9‐ and HCC827‐GRPs displaying epithelial‐like morphology versus shControl cells (scale bar indicate 200 μm). (c) qPCR analysis of mRNA expression of ZEB1 and BMI1, and miRNA expression of miR‐200c. (d) qPCR analysis of mRNA expression of vimentin and E‐cadherin. (e) qPCR analysis of mRNA expression of stem cell‐related factors. Data are normalized to beta actin (ACTB) expression. All values are average of triplicate experiments with error bars indicating SEM (ns, nonsignificant; *p < 0.05; **p < 0.01). (f) Western blotting analysis of ZEB1, CD133, and BMI1. GRP, gefitinib‐resistant persisters

To explore the role of ZEB1 in the maintenance of mesenchymal and CSC phenotypes in GRPs, we stably knocked down ZEB1 expression in PC9 and HCC827 cells using short‐hairpin RNAs (shRNAs) that specifically target ZEB1. The efficiency of knockdown of ZEB1 expression was confirmed by qPCR analysis (Figure 3(a)). Low ZEB1‐expressing cells, termed PC9‐ and HCC827‐shZEB1, were treated with gefitinib to obtain GRPs. As shown in Figure 3(b), the morphology of both PC9‐ and HCC827‐shZEB1 GRPs was more epithelial, while sh‐Control GRPs exhibited spindle‐shaped morphology reminiscent of the mesenchymal phenotype. Knockdown of ZEB1 led to upregulation of miR‐200c and significant reduction of BMI1 in PC9‐ and HCC827‐GRPs than in control cells (Figure 3(c)). Knockdown of ZEB1 also led to a decrease in the expression of vimentin and increase in E‐cadherin, accompanied by loss of mesenchymal morphology, suggesting that ZEB1 depletion reversed EMT in GRPs than in control cells (Figure 3(d)). Furthermore, CSC‐related gene signatures, such as CD133, ALDH1A1, Nanog, Oct4, CXCR4 and SOX2 were also reversed by ZEB1 knockdown in both PC9‐ and HCC827‐GRPs than in control cells (Figure 3(e)). Protein expression of ZEB1, CD133, and BMI1 was confirmed by western blot analysis (Figure 3(f)). The same result was obtained for transient ZEB1‐silenced GRPs using a small‐interfering RNA (siRNA) transfection system (Figure S2). These findings indicate that ZEB1 is required for the maintenance of mesenchymal and CSC phenotypes in GRPs in vitro.

### Sphere‐forming ability and in vivo tumorigenicity of GRPs are suppressed by ZEB1 knockdown

**FIGURE 4:**
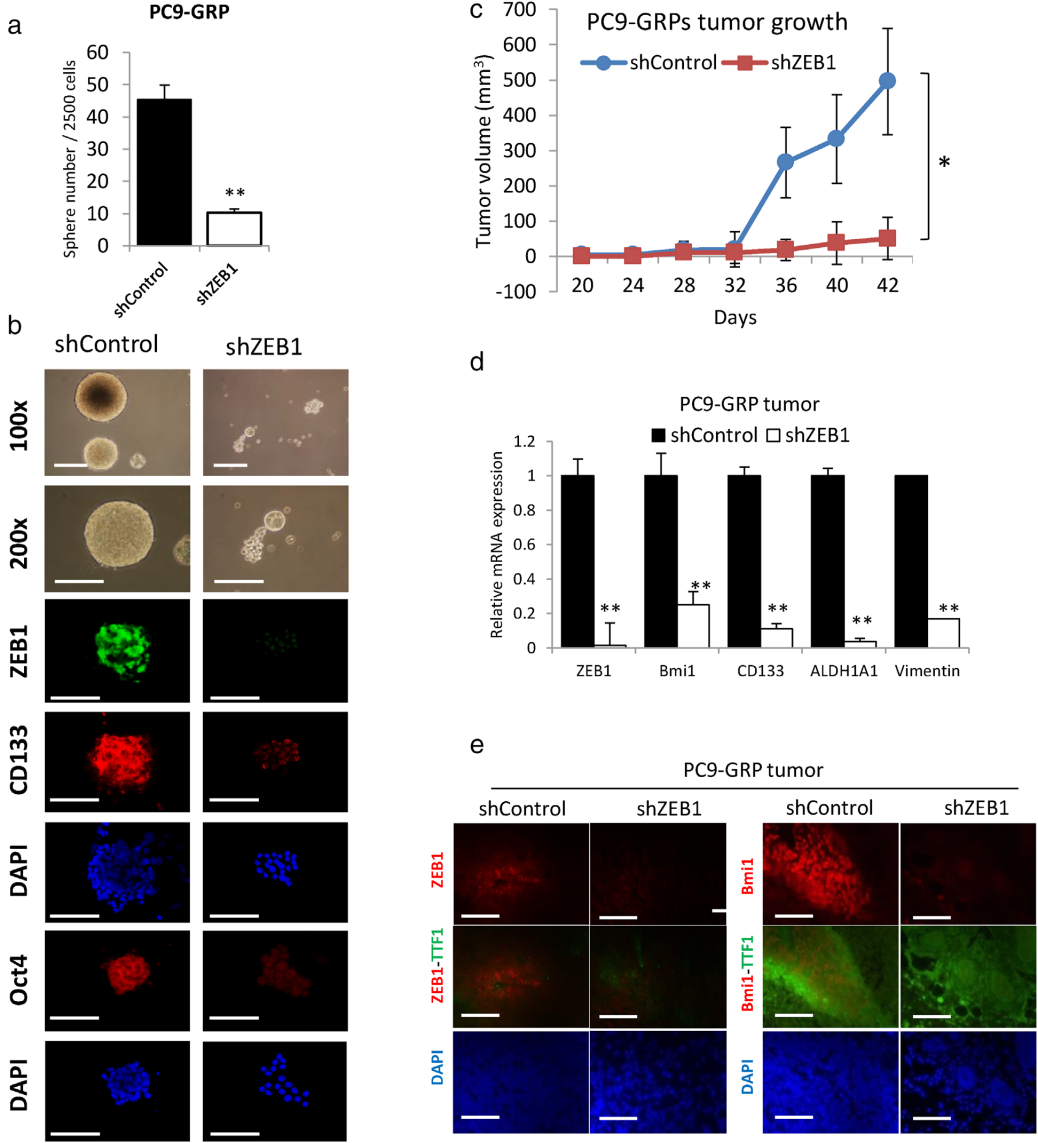
Knockdown of zinc‐finger E‐box‐binding homeobox 1 (ZEB1) reverses the in vitro self‐renewal and in vivo cancer stem cells (CSCs) phenotype of PC9‐GRPs. (a) Silencing of ZEB1 in PC9‐GRPs reduces the number of spheres than shControl (**p < 0.01). (b) Fluorescence immunohistochemistry analysis of spheres using antibodies against ZEB1, Oct4, or CD133. Cell nuclei are stained with DAPI (blue). Images are obtained on an Axioplan 2 imaging system with AxioVision software, scale bars indicate 200 μm. (c) Tumor volume analysis of PC9‐GRPs transduced with shControl or shZEB1. ZEB1‐silenced gefitinib‐resistant persisters (GRPs) showed significantly reduced tumor growth than shControl GRPs (*p < 0.05). (d) Quantitative real‐time PCR analysis of mRNA expression of ZEB1, BMI1, CD133, ALDH1A1 and vimentin. Data are normalized to beta actin (ACTB) expression and represent mean ± SEM for at least three tumors (*p < 0.05; **p < 0.01). (e) Double‐staining fluorescence immunohistochemistry with thyroid transcription factor 1 (TTF1) to analyze protein expression of ZEB1 and BMI1. Scale bars indicate 200 μm

To further evaluate the role of ZEB1 in the stemness of GRPs, tumor sphere‐forming assay reflecting the self‐renewal ability and in vivo tumorigenicity studies were performed. As shown in Figure 4(a), knockdown of ZEB1 significantly reduced the number of spheres in PC9‐GRPs than that in shControl. Immunofluorescence analysis indicated higher expression of ZEB1, CD133, and Oct4 in spheres of shControl‐GRPs, and lower expression of CD133 and Oct4 in ZEB1‐depleted GRPs‐spheres (Figure 4(b)). Next, we injected 100 GRPs of PC9‐shControl and PC9‐shZEB1 into both the flanks of NOG mice. Tumor growth of GRPs was significantly suppressed by knockdown of ZEB1 expression (Figure 4(c)). Silencing ZEB1 also significantly repressed the expression of BMI1, CD133, ALDH1A1, and mesenchymal marker vimentin than control (Figure 4(d)). Immunohistochemical findings revealed that BMI1, a known target of ZEB1, was suppressed by knockdown of ZEB1 in PC9‐GRPs tumors than in control (Figure 4(e)). These findings indicated that ZEB1 contributes to the self‐renewal ability and in vivo tumorigenicity in GRPs.

### Overexpression of ZEB1 induced EMT and gefitinib resistance and increased population of gefitinib‐resistant CSCs in vitro

**FIGURE 5:**
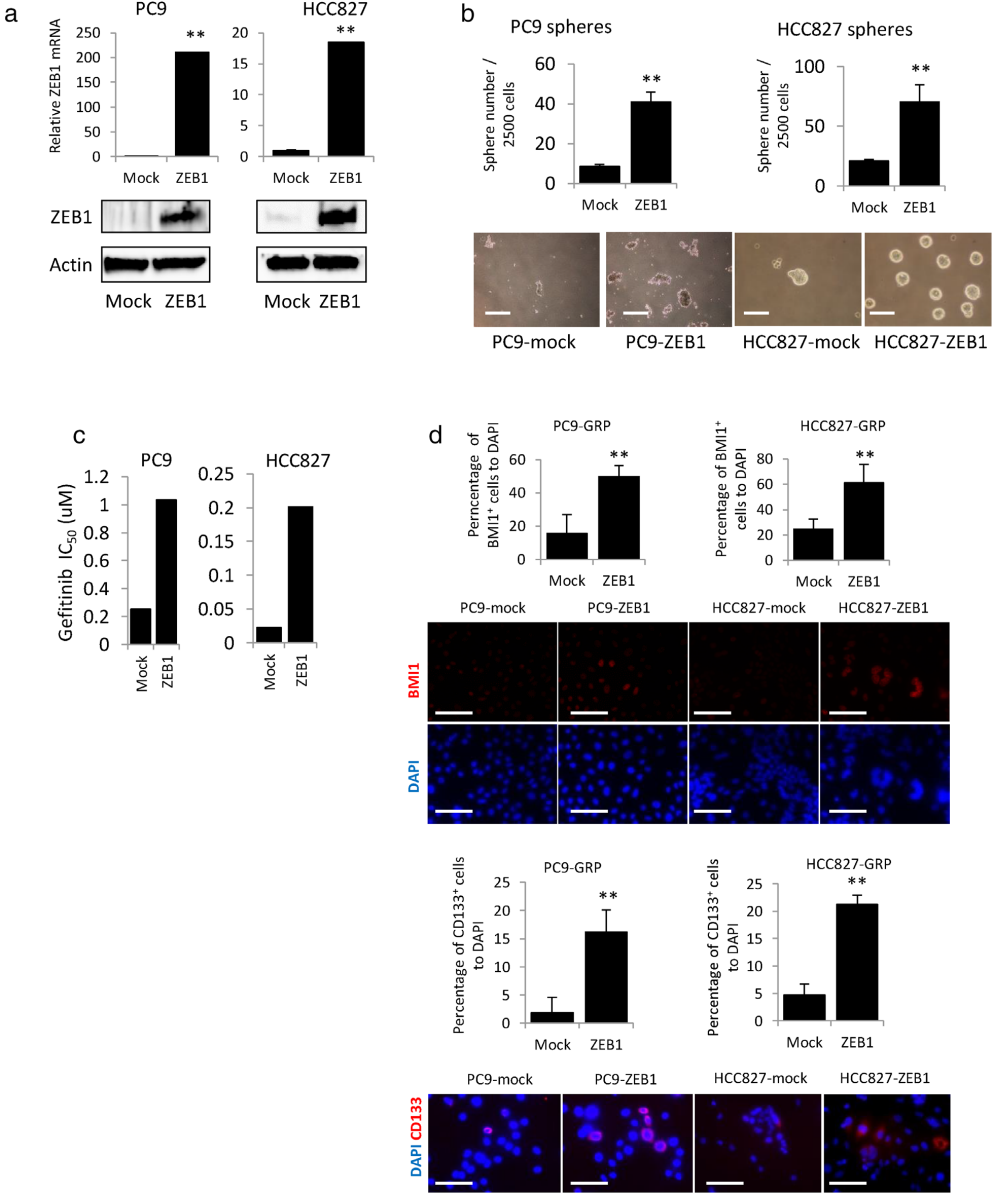
Overexpression of zinc‐finger E‐box‐binding homeobox 1 (ZEB1) increases self‐renewal ability and cancer stem cell (CSC) marker‐positive cells in PC9‐ and HCC827‐GRPs. (a) Quantitative real‐time PCR and western blotting analysis for confirming overexpression of ZEB1. (b) Sphere formation assay in PC9 and HCC827 cells overexpressing ZEB1 or mock (**p < 0.01, scale bars indicate 200 μm). (c) Quantification of half maximal inhibitory concentration (IC_50_) values for ZEB1‐overexpressing PC9 and HCC827 cells or mock treated with gradient concentrations of gefitinib. (d) Quantification of BMI1‐ and CD133‐positive PC9 and HCC827 cells overexpressing ZEB1 or mock treated with gefitinib. The number of CD133 and BMI1‐positive cells are counted and compared with DAPI numbers from each field. Data are shown as mean of positive cells percentage from five fields in each experiment. **p < 0.01, scale bars indicate 200 μm. GRP, gefitinib‐resistant persisters

To examine whether ZEB1 could induce EMT and enhance CSC features in *EGFR*‐mutant NSCLC, we transduced lentiviruses carrying the ZEB1 construct into PC9 and HCC827 cells. Overexpression of ZEB1 mRNA and protein was confirmed by qPCR and western blot analysis, respectively (Figure 5(a)). PC9 and HCC827 cells overexpressing ZEB1 (PC9‐ and HCC827‐ZEB1) exhibited spindle‐shaped morphology as compared to PC9‐ and HCC827‐mock cells. Furthermore, overexpression of ZEB1 suppressed the expression of E‐cadherin and increased expression of mesenchymal marker (Figure S3), reminiscent of EMT than mock cells. ZEB1‐overexpressing cells also had higher number of spheres than mock cells (Figure 5(b)). These findings indicated that overexpression of ZEB1 could induce EMT and enhance CSC features in *EGFR*‐mutant NSCLC cells.

Further, to investigate whether high ZEB1 expression could induce gefitinib resistance in vitro, PC9 and HCC827 cells overexpressing ZEB1 or mock were exposed to various concentrations of gefitinib to estimate IC_50_ values against gefitinib. As shown in Figure 5(c), overexpression of ZEB1 increased the IC_50_ of gefitinib in both PC9 and HCC827 cells than mock. Next, to explore the implications of high ZEB1 expression in the maintenance of GRPs with CSC features, cells overexpressing ZEB1 or mock were treated with high concentrations of gefitinib to obtain GRPs. Overexpression of ZEB1 significantly increased expression of BMI1‐ and CD133‐positive GRPs that could survive and remain viable after gefitinib treatment (Figure 5(d)). These findings indicate that ZEB1 is implicated in resistance to gefitinib and increases the population of gefitinib‐resistant lung CSCs in vitro.

### High ZEB1 expression induced resistance to gefitinib in vivo

**FIGURE 6:**
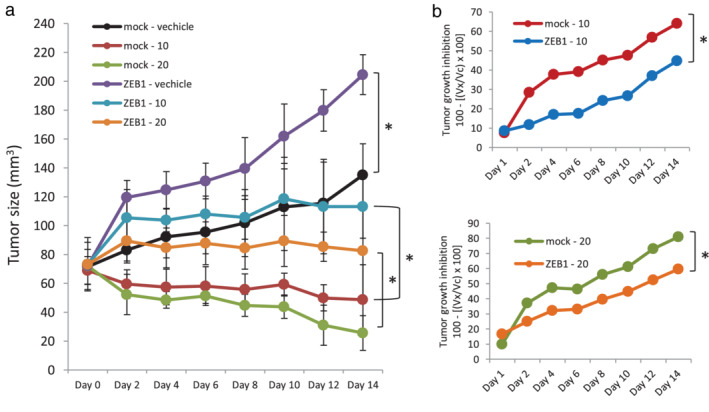
Overexpression of zinc‐finger E‐box‐binding homeobox 1 (ZEB1) increases tumor growth and induces resistance to gefitinib in vivo. (a) Comparison of tumor size for PC9‐mock and PC9‐ZEB1 tumors treated with vehicle or gefitinib (10 mg/ml or 20 mg/ml). (b) Quantification of antitumor activity of gefitinib is calculated for individual tumors as the percentage of tumor growth inhibition, according to the following formula: 100 – [(V_x_/V_c_) × 100], where V_x_ is the tumor volume for treated mice and V_c_ is tumor volume in the control group at a given time, *x*

To test the biological significance of high ZEB1 expression in conferring resistance to gefitinib in *EGFR*‐mutant NSCLC cells in vivo, PC9‐ZEB1 and PC9‐mock cells were implanted subcutaneously in NOG mice. When the average tumor volume reached 75 mm^3^, mice were divided into groups to receive vehicle, or 10 or 20 mg/kg of gefitinib via intraperitoneal injection (six times/week). The tumor size was measured during 14 days of treatment, and tumor volume was calculated every two days. Antitumor effect was estimated for individual tumors as percentage of tumor growth inhibition. As shown in Figure 6(a), in the vehicle‐treated group, tumor growth of PC9‐ZEB1 was significantly faster than that of PC9‐mock (*p* = 0.0028). In the gefitinib‐treated group, tumor growth was suppressed with gefitinib treatment to a lesser extent in mice bearing PC9‐ZEB1 tumors than those bearing PC9‐mock (Figure 6(a)), suggesting that the antitumor effect of both concentrations of gefitinib was significantly reduced in PC9‐ZEB1 mice than that in PC9‐mock mice (Figure 6(b)). These findings indicated a crucial role of ZEB1 in resistance to gefitinib in *EGFR*‐mutant NSCLC cells in vivo.

### ZEB1 is highly expressed in tumor specimens of NSCLC patients with acquired resistance to gefitinib

**FIGURE 7:**
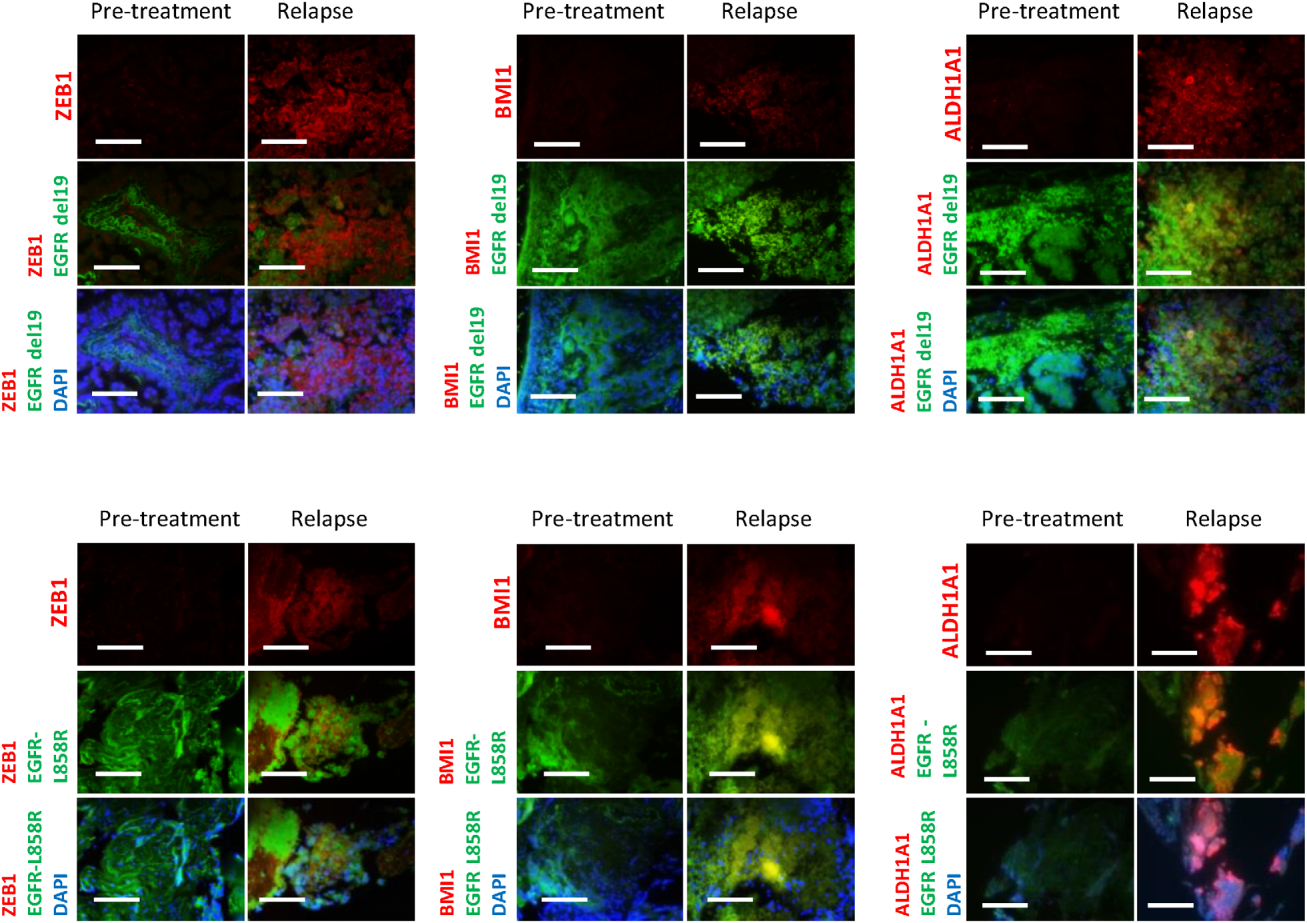
Specimens from lung cancer patients with acquired resistance to epidermal growth factor receptor‐tyrosine kinase inhibitors (EGFR‐TKIs) show increased expression of zinc‐finger E‐box‐binding homeobox 1 (ZEB1), BMI1, and ALDH1A1 proteins. Representative images of fluorescence immunohistochemistry staining of tumor samples from lung cancer patients pretreatment and after recurrence. Double staining using antibodies specific to either *EGFR*‐19del or *EGFR*‐L858R is performed to confirm the tumor area in the specimens. Scale bars indicate 200 μm. del, deletion

Finally, to confirm the in vitro and in vivo findings, we analyzed the expression of ZEB1, BMI1, and ALDH1A1 by immunohistochemistry in lung cancer specimens obtained from 20 NSCLC patients before treatment and after relapse during treatment with gefitinib. As shown in Table 1, in the pretreatment clinical samples, weak expression of ZEB1, BMI1, and ALDH1A1 was observed in 12, 8, and 8 samples, respectively. After acquiring resistance to gefitinib, 11 patients exhibited an *EGFR* T790M secondary mutation (Table 1), 17 cases exhibited moderate to strong expression of ZEB1 (Table 1, Figure 7), and 14 cases showed moderate to strong expression of BMI1 (Table 1, Figure 7). Finally, all samples with acquired resistance showed positive ALDH1A1 expression (Table 1, Figure 7). Taken together, these findings indicated that ZEB1, BMI1, and ALDH1A1 were highly expressed in tumor specimens from NSCLC patients with acquired resistance to gefitinib.

**TABLE 1:** Qualitative comparison of expression of ZEB1, BMI and ALDH1A1 in pretreated tumor and acquired resistance tumor specimens

Patients	Age (years)	*EGFR* mutation	T790M mutation in 2nd biopsy	Pretreated tumor			Acquired resistance tumor		
				ZEB1	BMI1	ALDH1A1	ZEB1	BMI1	ALDH1A1
Patient 1	65	19 del	+	+	+	+	++	+++	++
Patient 2	69	19 del	−	−	−	−	+++	++	++
Patient 3	65	L858R	−	−	−	−	++	+++	+++
Patient 4	71	19 del	−	−	−	−	−	+	++
Patient 5	74	L858R	−	+	−	−	++	++	++
Patient 6	58	19 del	+	+	+	−	+++	++	++
Patient 7	65	19 ins	+	−	−	−	++	+	+++
Patient 8	60	19 del	−	+	−	−	++	−	+++
Patient 9	60	L858R	−	+	−	−	+++	+	+++
Patient 10	70	L858R	+	+	−	−	+++	−	+++
Patient 11	75	L858R	+	+	+	+	+++	++	+++
Patient 12	75	L858R	+	−	−	+	+++	+++	+++
Patient 13	62	19 del	+	+	+	+	++	+++	+++
Patient 14	62	L858R	+	+	+	−	+	+++	+++
Patient 15	74	19 del	−	−	+	+	+	+++	++
Patient 16	65	L858R	−	−	+	+	+++	+++	+++
Patient 17	70	L858R	+	+	−	+	++	++	+++
Patient 18	78	L858R	−	+	−	−	+++	+++	+++
Patient 19	64	19 del	+	+	+	+	++	+++	+++
Patient 20	64	19 del	+	−	−	−	+++	+	++

Abbreviations: EGFR, epidermal growth factor receptor; del, deletion; ins, insertion.

## DISCUSSION

Recent studies suggest the involvement of EMT and CSCs in acquired resistance to EGFR‐TKIs in *EGFR*‐mutant NSCLC cells.^26^, ^27^, ^28^ However, the molecular mechanism for regulation of not only EMT but also CSCs in conferring resistance to EGFR‐TKIs remains unclear. ZEB1 is a crucial EMT inducer that directly suppresses the transcription of miR‐200c.^20^ Further, miR‐200c modulates the expression of BMI1, a known regulator of stem cell self‐renewal.^10^, ^25^ Interestingly, miR‐200c can also target ZEB1, and this loop linking ZEB1 and miR‐200c is important for mesenchymal or epithelial differentiation.^20^, ^29^ ZEB1 is involved in EMT‐mediated acquired resistance to gefitinib in *EGFR*‐mutant NSCLC cells,^22^ although the biological significance of ZEB1 in the lung CSCs contributing to acquired resistance to gefitinib has not been fully elucidated.

In this study, we treated PC9 and HCC827 NSCLC cell lines that harbor activating *EGFR* mutations with gefitinib. After gefitinib treatment for nine days, a subpopulation of cells survived, referred to as gefitinib‐resistant persisters (GRPs). These cells showed high expression of ZEB1 and BMI1 genes, and reduced expression of miR‐200c (ZEB1‐miR200c‐BMI1 axis). Moreover, mesenchymal markers, such as vimentin, fibronectin, N‐cadherin, and stem cell‐related factors were upregulated, along with loss of the epithelial marker E‐cadherin. In vivo, GRP tumor growth was faster and maintained the gene signature: ZEB1‐miR200c‐BMI1 axis; high expression of mesenchymal and stem cell‐related factors; and reduced expression of epithelial marker. Gefitinib‐resistant tumor (GRT), the remaining PC9 tumor in mice after treatment with gefitinib, also showed similar gene profiles. Furthermore, a stable knockdown of ZEB1 significantly attenuated the suppression of miR‐200c, which led to reduced expression of BMI1 genes and protein. Silencing of ZEB1 also reduced expression of stem cell‐related markers, including CD133, Oct4, SOX2, Nanog, CXCR4, ALDH1A1, and the mesenchymal marker vimentin, and restored E‐cadherin expression. Self‐renewal of GRPs, as shown by sphere formation capacity, was also decreased in ZEB1‐silenced GRPs, while in vivo tumorigenicity of GRPs was compromised. The overexpression of ZEB1 resulted in EMT and CSC phenotypes, and importantly, increased IC_50_ values of gefitinib and CD133‐ and BMI1‐positive GRPs in vitro. Overexpression of ZEB1 also induced resistance to gefitinib in vivo, as observed in the PC9 xenograft tumor model. Finally, the lung cancer specimens from patients with acquired resistance to *EGFR*‐TKI showed increased expression of ZEB1, BMI1, and ALDH1A, than in specimens before treatment. Taken together, the findings suggest that GRPs are enriched with EMT and CSC phenotype, and that ZEB1 plays an important role in maintaining not only EMT but also CSC phenotype in GRPs via the miR‐200c and BMI1 pathways.

Shien et al. established clonal gefitinib‐resistant NSCLC cells and demonstrated that few resistant cells with EMT signatures exhibited CSC properties.^30^ Of those, clones of gefitinib‐resistant HCC827 cells showed high expression of ZEB1 and silencing of miR‐200c.^30^ They also demonstrated that silencing of miR‐200c was associated with increased expression of LIN28B, which regulates stem cell self‐renewal and miR‐200c activity. The LIN28B axis plays an important role in cells with acquired resistance to EGFR‐TKIs that harbor EMT features.^31^ A recent study by Weng et al. also established osimertinib‐resistant H1975 cells with mesenchymal features, and demonstrated that these cells were capable of forming larger spheroids and showed high expression of ZEB1.^28^ Although knockdown or overexpression of ZEB1 was not performed, these previous reports strongly support our hypothesis that ZEB1 plays a crucial role in EGFR‐TKI–resistant lung CSCs with EMT features.

Cufi et al. demonstrated that EMT‐driven resistance to erlotinib could be overcome by reversing the high‐ZEB1/low‐miR200c signature in vivo.^32^ Silibinin, a potent natural agent derived from dried fruits of milk thistle (*Silybum marianum*),^33^ suppressed mesenchymal gene expression including ZEB1 and reversed suppression of miR‐200c signature in erlotinib‐refractory tumors; and, complete abrogation of tumor growth was observed after cotreatment with erlotinib and silibinin. Consistent with the findings of the preclinical study using silibinin, silencing of ZEB1 restored miR‐200c expression in GRP tumor model in our study, along with reduced tumor incidence and slower tumor growth in vivo. These findings suggest that the combination of EGFR‐TKI with a ZEB1‐targeting approach would be effective in preventing EMT and CSC‐mediated resistance to EGFR‐TKI.

The expression of ZEB1 in cancer cells from NSCLC patients with acquired resistance to EGFR‐TKI has been previously investigated.^22^ However, to the best of our knowledge, no study has examined the expression of both ZEB1 and BMI1 in lung cancer specimens from patients with acquired resistance to EGFR‐TKI. The current study revealed that ZEB1 protein expression was higher in cancer cells than in before‐treatment cancer cells. This is consistent with the in vitro findings of our study using NSCLC cell lines, in which ZEB1 expression was significantly upregulated in the GRP of PC9 and HCC827 than in parental cells. Lung tumor specimens from NSCLC patients with acquired resistance to EGFR‐TKI also showed high BMI1 protein expression, suggesting that the ZEB1–BMI1 axis exists in the clinical setting. Therefore, ZEB1 and BMI1 expression may be useful in monitoring resistance to EGFR‐TKI in NSCLC patients who were treated with EGFR‐TKI.

There are several limitations of this study. First, we did not use NSCLC cell lines with *EGFR L858R* mutation, although we showed that ZEB1 was highly expressed in tumor specimens from NSCLC patients with *EGFR L858R* mutation after gefitinib resistance. Second, osimertinib, a third‐generation EGFR‐TKI, is also currently used for the first‐line treatment of *EGFR*‐mutant NSCLC patients as the standard of care. To address the biological significance of ZEB1 in lung cancer stem cells resistant to osimertinib in this clinical setting, further studies to establish osimertinib‐resistant persisters from *EGFR*‐mutant NSCLC cells are necessary. In addition, relapsed tumor tissues from *EGFR*‐mutant NSCLC patients after first‐line treatment with osimertinib are required.

To the best of our knowledge, this is the first study to reveal the central role of ZEB1 in conferring resistance to gefitinib by not only inducing EMT but also by maintaining the CSC phenotype in NSCLC by regulating expression of miR‐200c and BMI1, along with activating *EGFR* mutations. We propose a combination of EGFR‐TKI with ZEB1‐targeting therapy to prevent the survival of NSCLC cells that show EMT and CSC phenotypes. Further studies using ZEB1‐targeted therapy, such as silibinin, would be required to verify the results of this study.

## CONFLICT OF INTEREST

Kazuhisa Takahashi and Fumiyuki Takahashi received research funding from Chugai Pharm, Ono Pharma, Taiho Pharm, Nippon Boehringer Ingelheim, AstraZeneca, Pfizer, MSD, and Lilly Japan, outside the submitted work. The remaining authors declare that they have no conflicts of interest relevant to the subject of this manuscript.

## Supporting information


**Appendix S1**: Supporting information

## References

[1] Costa DB , Halmos B , Kumar A , Schumer ST , Huberman MS , et al. BIM mediates EGFR tyrosine kinase inhibitor‐induced apoptosis in lung cancers with oncogenic EGFR mutations. PLoS Med. 2007;4:1669–80. 17973572 10.1371/journal.pmed.0040315 PMC2043012

[2] Yun CH , Mengwasser KE , Toms AV , Woo MS , Greulich H , Wong KK , et al. The T790M mutation in EGFR kinase causes drug resistance by increasing the affinity for ATP. Proc Natl Acad Sci U S A. 2008;105:2070–5. 18227510 10.1073/pnas.0709662105 PMC2538882

[3] Pao W , Miller VA , Politi KA , Riely GJ , Somwar R , Zakowski MF , et al. Acquired resistance of lung adenocarcinomas to gefitinib or erlotinib is associated with a second mutation in the EGFR kinase domain. PLoS Med. 2005;2:e73. 15737014 10.1371/journal.pmed.0020073 PMC549606

[4] Engelman JA , Zejnullahu K , Mitsudomi T , Song Y , Hyland C , Park JO , et al. MET amplification leads to gefitinib resistance in lung cancer by activating ERBB3 signaling. Science. 2007;316:1039–43. 17463250 10.1126/science.1141478

[5] Bean J , Brennan C , Shih JY , Riely G , Viale A , Wang L , et al. MET amplification occurs with or without T790M mutations in EGFR mutant lung tumors with acquired resistance to gefitinib or erlotinib. Proc Natl Acad Sci U S A. 2007;104:20932–7. 18093943 10.1073/pnas.0710370104 PMC2409244

[6] Yano S , Yamada T , Takeuchi S , Tachibana K , Minami Y , Yatabe Y , et al. Hepatocyte growth factor expression in EGFR mutant lung cancer with intrinsic and acquired resistance to tyrosine kinase inhibitors in a Japanese cohort. J Thorac Oncol. 2011;6:2011–7. 22052230 10.1097/JTO.0b013e31823ab0dd

[7] Sequist LV , Waltman BA , Dias‐Santagata D , Digumarthy S , Turke AB , Fidias P , et al. Genotypic and histological evolution of lung cancers acquiring resistance to EGFR inhibitors. Sci Transl Med. 2011;3:75ra26. 10.1126/scitranslmed.3002003 PMC3132801 21430269

[8] Takezawa K , Pirazzoli V , Arcila ME , Nebhan CA , Song X , de Stanchina E , et al. HER2 amplification: A potential mechanism of acquired resistance to EGFR inhibition in EGFR‐mutant lung cancers that lack the second‐site EGFRT790M mutation. Cancer Discov. 2012;2:922–33. 22956644 10.1158/2159-8290.CD-12-0108 PMC3473100

[9] Eramo A , Lotti F , Sette G , Pilozzi E , Biffoni M , di Virgilio A , et al. Identification and expansion of the tumorigenic lung cancer stem cell population. Cell Death Differ. 2008;15:504–14. 18049477 10.1038/sj.cdd.4402283

[10] Zacharek SJ , Fillmore CM , Lau AN , Gludish DW , Chou A , Ho JWK , et al. Lung stem cell self‐renewal relies on BMI1‐dependent control of expression at imprinted loci. Cell Stem Cell. 2011;9:272–81. 21885022 10.1016/j.stem.2011.07.007 PMC3167236

[11] Jiang T , Collins BJ , Jin N , Watkins DN , Brock MV , Matsui W , et al. Achaete‐scute complex homologue 1 regulates tumor‐initiating capacity in human small cell lung cancer. Cancer Res. 2009;69:845–54. 19176379 10.1158/0008-5472.CAN-08-2762 PMC2919317

[12] Chen YC , Hsu HS , Chen YW , Tsai TH , How CK , et al. Oct‐4 expression maintained cancer stem‐like properties in lung cancer‐derived CD133‐positive cells. PLoS One. 2008;3:e2637. 18612434 10.1371/journal.pone.0002637 PMC2440807

[13] Jung MJ , Rho JK , Kim YM , Jung JE , Jin YB , Ko YG , et al. Upregulation of CXCR4 is functionally crucial for maintenance of stemness in drug‐resistant non‐small cell lung cancer cells. Oncogene. 2013;32:209–21. 22370645 10.1038/onc.2012.37

[14] Cheng X , Chen H . Tumor heterogeneity and resistance to EGFR‐targeted therapy in advanced nonsmall cell lung cancer: Challenges and perspectives. Onco Targets Ther. 2014;7:1689–704. 25285017 10.2147/OTT.S66502 PMC4181629

[15] Mani SA , Guo W , Liao MJ , Eaton EN , Ayyanan A , Zhou AY , et al. The epithelial‐mesenchymal transition generates cells with properties of stem cells. Cell. 2008;133:704–15. 18485877 10.1016/j.cell.2008.03.027 PMC2728032

[16] Chang TH , Tsai MF , Su KY , Wu SG , Huang CP , Yu SL , et al. Slug confers resistance to the epidermal growth factor receptor tyrosine kinase inhibitor. Am J Respir Crit Care Med. 2011;183:1071–9. 21037017 10.1164/rccm.201009-1440OC

[17] Ren S , Su C , Wang Z , Li J , Fan L , Li B , et al. Epithelial phenotype as a predictive marker for response to EGFR‐TKIs in non‐small cell lung cancer patients with wild‐type EGFR. Int J Cancer. 2014;135:2962–71. 24771540 10.1002/ijc.28925

[18] Li L , Han R , Xiao H , Lin C , Wang Y , Liu H , et al. Metformin sensitizes EGFR‐TKI‐resistant human lung cancer cells in vitro and in vivo through inhibition of IL‐6 signaling and EMT reversal. Clin Cancer Res. 2014;20:2714–26. 24644001 10.1158/1078-0432.CCR-13-2613

[19] Ju L , Zhou C . Integrin beta 1 enhances the epithelial‐mesenchymal transition in association with gefitinib resistance of non‐small cell lung cancer. Cancer Biomark. 2013;13:329–36. 24440972 10.3233/CBM-130362 PMC12928299

[20] Burk U , Schubert J , Wellner U , Schmalhofer O , Vincan E , Spaderna S , et al. A reciprocal repression between ZEB1 and members of the miR‐200 family promotes EMT and invasion in cancer cells. EMBO Rep. 2008;9:582–9. 18483486 10.1038/embor.2008.74 PMC2396950

[21] Wellner U , Schubert J , Burk UC , Schmalhofer O , Zhu F , Sonntag A , et al. The EMT‐activator ZEB1 promotes tumorigenicity by repressing stemness‐inhibiting microRNAs. Nat Cell Biol. 2009;11:1487–95. 19935649 10.1038/ncb1998

[22] Yoshida T , Song L , Bai Y , Kinose F , Li J , Ohaegbulam KC , et al. ZEB1 mediates acquired resistance to the epidermal growth factor receptor‐tyrosine kinase inhibitors in non‐small cell lung cancer. PLoS One. 2016;11:e0147344. 26789630 10.1371/journal.pone.0147344 PMC4720447

[23] Pan H , Jiang T , Cheng N , Wang Q , Ren S , Li X , et al. Long non‐coding RNA BC087858 induces non‐T790M mutation acquired resistance to EGFR‐TKIs by activating PI3K/AKT and MEK/ERK pathways and EMT in non‐small‐cell lung cancer. Oncotarget. 2016;7:49948–60. 27409677 10.18632/oncotarget.10521 PMC5226560

[24] Murakami A , Takahashi F , Nurwidya F , Kobayashi I , Minakata K , Hashimoto M , et al. Hypoxia increases gefitinib‐resistant lung cancer stem cells through the activation of insulin‐like growth factor 1 receptor. PLoS One. 2014;9:e86459. 24489728 10.1371/journal.pone.0086459 PMC3904884

[25] Shimono Y , Zabala M , Cho RW , Lobo N , Dalerba P , Qian D , et al. Downregulation of miRNA‐200c links breast cancer stem cells with normal stem cells. Cell. 2009;138:592–603. 19665978 10.1016/j.cell.2009.07.011 PMC2731699

[26] Yao W , Wang L , Huang H , Li X , Wang P , Mi K , et al. All‐trans retinoic acid reduces cancer stem cell‐like cell‐mediated resistance to gefitinib in NSCLC adenocarcinoma cells. BMC Cancer. 2020;20:315. 32293355 10.1186/s12885-020-06818-0 PMC7161137

[27] Si J , Ma Y , Bi JW , Xiong Y , Lv C , Li S , et al. Shisa3 brakes resistance to EGFR‐TKIs in lung adenocarcinoma by suppressing cancer stem cell properties. J Exp Clin Cancer Res. 2019;38:481. 31801598 10.1186/s13046-019-1486-3 PMC6894286

[28] Weng CH , Chen LY , Lin YC , Shih JY , Lin YC , Tseng RY , et al. Epithelial‐mesenchymal transition (EMT) beyond EGFR mutations per se is a common mechanism for acquired resistance to EGFR TKI. Oncogene. 2019;38:455–68. 30111817 10.1038/s41388-018-0454-2

[29] Fukuda S , Nishida‐Fukuda H , Nanba D , Nakashiro KI , Nakayama H , Kubota H , et al. Reversible interconversion and maintenance of mammary epithelial cell characteristics by the ligand‐regulated EGFR system. Sci Rep. 2016;6:20209. 26831618 10.1038/srep20209 PMC4735799

[30] Shien K , Toyooka S , Yamamoto H , Soh J , Jida M , Thu KL , et al. Acquired resistance to EGFR inhibitors is associated with a manifestation of stem cell‐like properties in cancer cells. Cancer Res. 2013;73:3051–61. 23542356 10.1158/0008-5472.CAN-12-4136 PMC4506773

[31] Sato H , Shien K , Tomida S , Okayasu K , Suzawa K , Hashida S , et al. Targeting the miR‐200c/LIN28B axis in acquired EGFR‐TKI resistance non‐small cell lung cancer cells harboring EMT features. Sci Rep. 2017;7:40847. 28084458 10.1038/srep40847 PMC5233972

[32] Cufi S , Bonavia R , Vazquez‐Martin A , Oliveras‐Ferraros C , Corominas‐Faja B , Cuyàs E , et al. Silibinin suppresses EMT‐driven erlotinib resistance by reversing the high miR‐21/low miR‐200c signature in vivo. Sci Rep. 2013;3:2459. 23963283 10.1038/srep02459 PMC3748425

[33] Tamayo C , Diamond S . Review of clinical trials evaluating safety and efficacy of milk thistle (*Silybum marianum* [L.] Gaertn.). Integr Cancer Ther. 2007;6:146–57. 17548793 10.1177/1534735407301942

