# Supplementary material for: Zinc‐finger E‐box‐binding homeobox 1 (ZEB1) plays a crucial role in the maintenance of lung cancer stem cells resistant to gefitinib
Source: Thorac Cancer. 2021 Mar 25;12(10):1536–48. doi: 10.1111/1759-7714.13937 (PMC8107025; doi:10.1111/1759-7714.13937)
Supplement: Supplementary file 1 — Appendix S1: Supporting information [file TCA-12-1536-s001.docx]

**Supporting information**

**ZEB1 plays a crucial role in the maintenance of lung cancer stem cells resistant to gefitinib**

Fariz Nurwidya, Fumiyuki Takahashi, Wira Winardi, Ken Tajima, Yoichiro Mitsuishi, Akiko Murakami, Isao Kobayashi, Takeshi Nara, Muneaki Hashimoto, Motoyasu Kato, Moulid Hidayat, Kentaro Suina, Daisuke Hayakawa, Tetsuhiko Asao, Ryo Ko, Takehito Shukuya, Toshifumi Yae, Naoko Shimada, Yasuko Yoshioka, Shinichi Sasaki, Kazuhisa Takahashi

**Supporting methods**

**Morphological analysis by light microscopy**

PC9 and HCC827 cells were grown to 70% confluence and visualized at 200× magnification with an Olympus light microscope (Olympus, Japan). Digital images of the cells were randomly obtained and compared for morphological characteristics consistent with epithelial-mesenchymal transition (EMT).

**Western blotting**

Briefly, equivalent amounts of proteins were separated by SDS-PAGE and transferred to a Trans-blot Turbo mini-format 0.2 µm polyvinylidene difluoride membrane (Bio-Rad, Hercules, CA). The membrane was probed with a primary antibody followed by a horseradish peroxidase-conjugated secondary antibody. The complete list of antibodies used is described in the following section. The bands were visualized with Clarity^TM^ Western ECL Substrate (Bio-Rad, Hercules, CA), and images of blotted patterns were obtained using ImageQuant LAS4000mini (GE Healthcare, Pittsburg, PA).

**Antibodies**

For western blotting, we used the following antibodies: ZEB1, 1:100 dilution and BMI1 (D42B3), 1:100 dilution (Cell Signaling, Danvers, MA); CD133, 1:100 dilution (Miltenyi Biotec, Auburn, CA); E-cadherin (sc-21791), 1:500 dilution (Santa Cruz Biotechnology, Santa Cruz, CA); Vimentin, 1:200 dilution (BD Pharmingen, San Jose, CA); and β-actin, 1:3000 (Sigma-Aldrich, St Louis, MO).

For *in vitro* immunofluorescence assay, we used the following antibodies: ZEB1 (sc-25388), 1:500 dilution (Santa Cruz Technology, Santa Cruz, CA); CD133, 1:50 dilution (Miltenyi Biotec, Auburn, CA); and Oct4 (sc-5279), 1:100 dilution (Santa Cruz Biotechnology, Santa Cruz, CA). *In vivo* florescence immunohistochemistry antibodies used were as follows: ZEB1, 1:100 dilution (Novus Biologicals, Littleton, CO); BMI1, 1:200 dilution (LSBio, Seattle, WA); and TTF1 (D2E8) rabbit mAb, 1:100 dilution (Cell Signaling, Danvers, MA).

Secondary antibodies used for *in vitro* immunofluorescence, and fluorescence immunohistochemistry *in vivo* and clinical specimens were Alexa Fluor^®^ 488 goat anti-rabbit IgG and Alexa Fluor^®^ 594 goat anti-mouse IgG (Invitrogen, Carlsbad, CA, USA).

**List of quantitative real-time PCR (qPCR) primers**

ZEB1 Forward, 5’-AACCCAACTTGAACGTCACA-3’

Reverse, 5’-ATTACACCCAGACTGCGTCA-3’

BMI1 Forward, 5’-GCTGCCAATGGCTCTAATGAA-3’

Reverse, 5’-TGCTGGGCATCGTAAGTATCTT-3’

E-cadherin Forward, 5’-CACGGTAACCGATCAGAATG-3’

Reverse, 5’-ACCTCCATCACAGAGGTTCC-3

Vimentin Forward, 5’-AATTGCAGGAGGAGATGCTT-3’

Reverse, 5’-GAGACGCATTGTCAACATCC-3’

Fibronectin Forward, 5’-GAAGCCGAGGTTTTAACTGC-3’

Reverse, 5’-ACCCACTCGGTAAGTGTTCC-3’

CD133 Forward, 5’-GGCCCAGTACAACACTACCAA-3’

Reverse, 5’-CGCCTCCTAGCACTGAATTGATA-3’

Oct4 Forward, 5’-GAGTGAGAGGCAACCTGGAG-3’

Reverse, 5’-GCCGGTTACAGAACCACACT-3’

Sox2 Forward, 5’-TACAGCATGTCCTACTCGCAG-3’

Reverse, 5’-GAGGAAGAGGTAACCACAGGG-3’

Nanog Forward, 5’-TTTGTGGGCCTGAAGAAAACT-3’

Reverse, 5’-AGGGCTGTCCTGAATAAGCAG-3’

CXCR4 Forward, 5’-ACGCCACCAACAGTCAGAG-3’

Reverse, 5’-AGTCGGGAATAGTCAGCAGGA-3’

ALDH1A1 Forward, 5’-GCACGCCAGACTTACCTGTC-3’

Reverse, 5’-CCTCCTCAGTTGCAGGATTAAAG-3’

ACTB Forward, 5’-CTCTTCCAGCCTTCCTTCCT-3’

Reverse, 5’-AGCACTGTGTTGGCGTACAG-3’

**RNA interference**

Small interfering RNAs (siRNAs) targeting *ZEB1* (Stealth Select RNAi siRNA) were custom synthesized by Invitrogen. A negative control was purchased from Invitrogen. To exclude off-target effects, PC9 cells were transfected with 2 different *ZEB1*-specific siRNAs and 1 nonspecific control siRNA using Lipofectamine RNAiMAX (Invitrogen), according to the manufacturer’s instructions. The cells were detached and resuspended in complete growth medium without antibiotics, and then plated in each well. RNAi duplex and Lipofectamine RNAiMAX were mixed in Opti-MEM®I (Gibco) reduced serum medium and incubated for 15 min at room temperature. RNAi duplex-Lipofectamine™ RNAiMAX complexes were added to the wells containing cells. The cells were then incubated for 24 h at 37°C, followed by gefitinib treatment. The sequences of the siRNA targeting *ZEB1* were as follows:

Stealth *ZEB1* oligo #1: 5-AAACAGAGGACUCAGGCUUCUCAGC-3

Stealth *ZEB1* oligo #2: 5-UUAAGCAUGGAACACUGUUCUGGUC-3

**Fluorescence immunohistochemistry (F-IHC)**

Paraffin-fixed slides were subjected to the following steps: deparafinization, heat-mediated antigen retrieval in 10 mM citric acid buffer (pH 6.0), blocking with 5% goat serum in PBST, and F-IHC staining with ZEB1 antibody (1:100 dilution; Novus Biologicals, Littleton, CO), BMI1 antibody (1:200 dilution; LSBio, Seattle, WA), and ALDH1A1 antibody (1:75 dilution; Abcam, Cambridge, UK). Double-staining was performed using antibodies specific to *EGFR*-19del (D6B6), *EGFR*-L858R (43B2), or thyroid transcription factor 1 (TTF1) (1:100 dilutions; Cell Signaling, Danvers, MA) depending on the *EGFR* mutational status of the patient. Percentages of ZEB1-, BMI1-, or ALDH1A1-positive cells to DAPI were graded as follows: -/negative expression (0–5%); +/weak expression (5.1–33%); ++/moderate expression (33.1–66%); +++/strong expression (66.1–100%).

**Supporting figures**


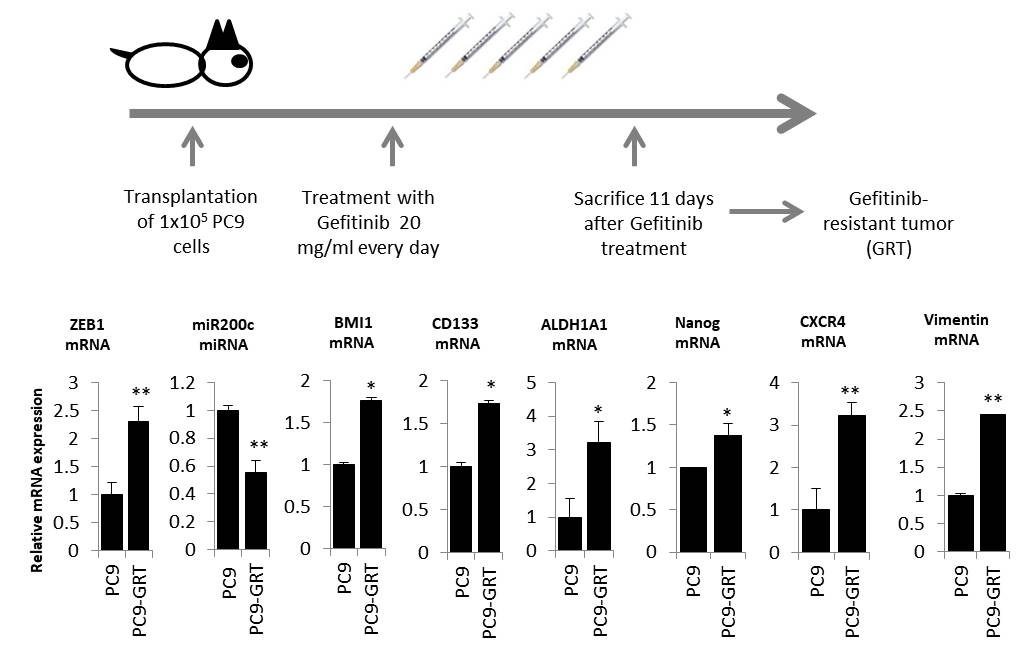


**Figure S1** Establishment of gefitinib-resistant tumor (GRT) of PC9 cells in NOG mice

Schematic representation of generation of PC9-GRTs (upper panel). PC9, 1x10^5^ cells, were transplanted into NOG mice followed by intraperitoneal injection of gefitinib 20mg/ml every day. Mice were sacrificed 11 days after gefitinib treatment and the gefitinib-resistant tumors (GRT) were subjected to RNA extraction and qPCR for expression of ZEB1, miR-200c, and BMI1, along with CSC-related genes and vimentin (lower panel). Data are normalized to *ACTB* expression and represent mean ± SEM (**P* < 0.05, ***P* < 0.01). *CSC*, cancer stem cells.


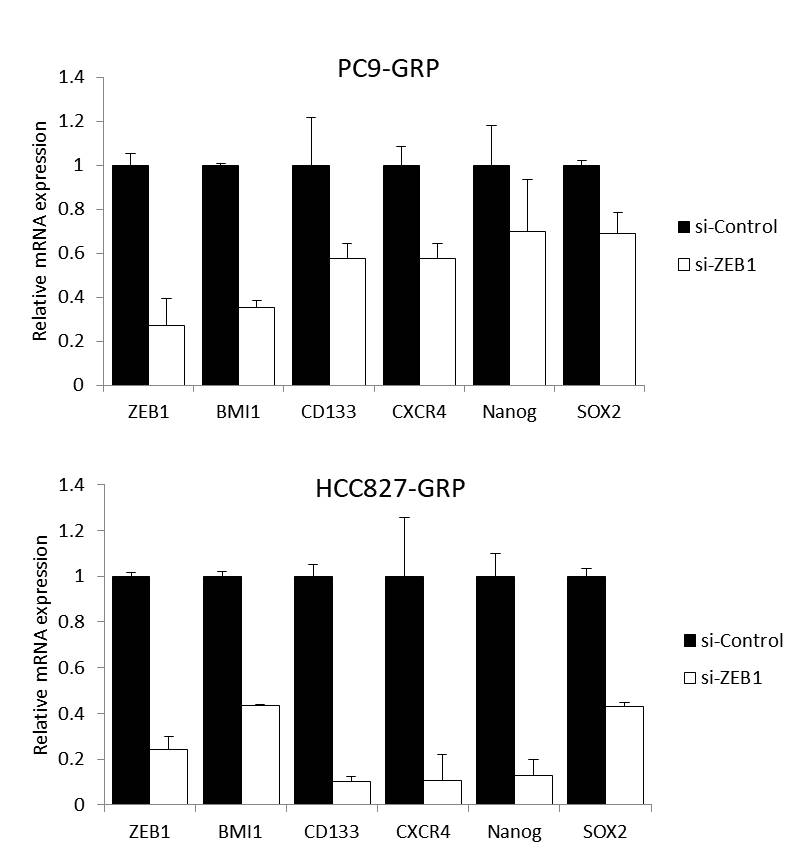


**Figure S2** Silencing of *ZEB1* by siRNA reduces expression of CSC genes in PC9- and HCC827-GRPs. The mRNA expression of ZEB1, BMI1, CD133, CXCR4, Nanog, and SOX2 is evaluated by quantitative real-time PCR. Data are normalized to *ACTB* expression. All values are the average of triplicate experiments with error bars indicating SEM.


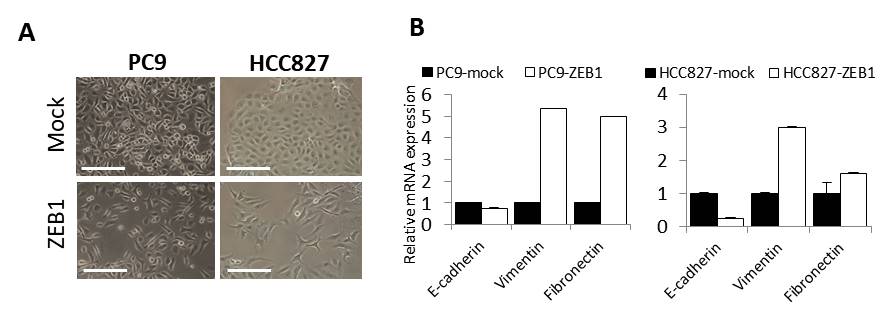


**Figure S3** Overexpression of *ZEB1* induces EMT in PC9 and HCC827 cells. (**A**) Overexpression of *ZEB1* results in a spindle-shaped and irregular morphology. Cell morphology is captured by light microscopy, with 200× magnification, scale bars indicate 200 μm. (**B**) The mRNA expression levels of E-cadherin, vimentin, and fibronectin are evaluated by quantitative real-time PCR. Data are normalized to *ACTB* expression and represent mean ± SEM of triplicate experiments.
